# The effect of predation risk on spermatophore deposition rate of the eriophyoid mite, *Aculops allotrichus*

**DOI:** 10.1007/s10493-015-9998-9

**Published:** 2015-12-21

**Authors:** Katarzyna Michalska

**Affiliations:** Department of Applied Entomology, Warsaw University of Life Sciences, Nowoursnowska 159, 02-776 Warsaw, Poland

**Keywords:** *Aculops allotrichus*, *Amblyseius swirskii*, Eriophyoidea, Phytoseiidae, Predation risk, Spermatophore deposition

## Abstract

Eriophyoids are minute herbivores in which males deposit spermatophores on a substrate while females, independent of the presence of males, pick up sperm (sex dissociation). Their most dangerous enemies are phytoseiid mites. Eriophyoids can successfully avoid the predation by, e.g., forming galls in which they live, by inhabiting narrow spaces on plants, or by climbing up leaf trichomes for the time of quiescence. All these behaviours, however, are fixed and independent of the actual risk of predation. The aim of this study was to examine whether eriophyoids can respond to the cues of predation risk and how this could affect their spermatophore deposition rate. *Aculops allotrichus* is a vagrant eriophyoid which inhabits leaves of the black locust tree, *Robinia pseudoacacia*. On leaf arenas with injured conspecifics (pierced with a fine needle which simulated the attack of phytoseiids), single males of *Ac. allotrichus* deposited a similar number of spermatophores as on control, ‘clean’ leaves. They did not respond to the cues left by the non-enemy, yeast-fed acarid mite *Tyrophagus putrescentiae* either. However, they deposited significantly fewer spermatophores on leaf arenas previously exposed to the presence of the eriophyoid-fed phytoseiid mite *Amblyseius swirskii*. This is a first report indicating that eriophyoids can respond to the cues left by predators and change their reproductive activity accordingly. The ultimate and proximate factors that may influence the behaviour of *Ac. allotrichus* males are discussed.

## Introduction

Generally, when searching for mates, courting or copulating, males and females tend to become more conspicuous and vulnerable to predators (Gwynne 1989; Rowe 1994; Kemp 2012). Thus, in pairing taxa, the mating frequency, intensity and duration of courtship or female guarding often diminish under an elevated predation risk (Sih et al. 1990; Acharya and McNeil 1998; Koga et al. 1998; Maier et al. 2000; Taylor et al. 2005; Oku and Yano 2008). This also applies to species reproducing via spermatophores deposited on a substrate, e.g., the salamander *Desmognahtus ochrophaeus* Copein males, which inhibited courtship behaviour and spermatophore deposition in the presence of a predator (Uzendoski et al. 1993).

To assess the risk of predation, a prey can use cues produced by a predator (e.g., kairomones emitted from the predator’s body, faeces, urine or other exudates) or indirect cues of predation, e.g., alarm pheromones produced by conspecifics or cues from dead conspecifics (Thorson et al. 1998; Grostal and Dicke 2000). Recent findings reveal that phytophagous mites, such as spider mites, can also respond to predation risk by avoiding leaf patches with cues of phytoseiid mites or other predatory arthropods and/or injured conspecifics on them (Kriesch and Dicke 1997; Grostal and Dicke 1999, 2000; Magalhães et al. 2002; Oku et al. 2003; Choh and Takabayashi 2007; Bowler et al. 2013; Otsuki and Yano 2014). Moreover, they also reduce oviposition when under elevated predation risk (Grostal and Dicke 1999, 2000; Oku et al. 2004; Škaloudová et al. 2007; Fernández-Ferrari and Schausberger 2013; Hackl and Schausberger 2014).

Eriophyoids are tiny (0.1–0.3 mm long), four-legged herbivorous mites, among which several species are serious pests of crop plants. By feeding, they can cause growth distortion, chlorosis and necrosis of plant tissue. They can also transmit plant viruses and pathogenic fungi (Lindquist et al. 1996; de Lillo and Skoracka 2010; Gamliel-Atinsky et al. 2010).

Phytoseiid mites are the most dangerous predators of eriophyoids, and are also used in practice to control eriophyoid pests (Van Leeuwen et al. 2010; Gadino and Walton 2012; Maoz et al. 2014). They are much bigger and faster than eriophyoids, and can detect the presence of the herbivores from long distances due to odours emitted from injured plants (Dicke et al. 1988; Sabelis and Bruin 1996; Melo et al. 2011).

Eriophyoids can successfully avoid phytoseiids by inhabiting galls or narrow spaces on plants, which the predatory mites are unable to enter (Sabelis and Bruin 1996). Vagrant species can also temporarily hide from predators. In *Rhinophytoptus concinnus* Liro from elm tree (Michalska 2003) and *Aculus comatus* (Nalepa) from filbert (Krantz 1973), juveniles climb up leaf trichomes and spend their quiescence period on the trichome tips. All these behaviours, however, are fixed, i.e., take place regardless of whether predators are present on plants or not (Michalska et al. 2010).

The aim of this study was to examine whether eriophyoids respond to direct and indirect cues of predation risk and whether this might affect their spermatophore output. Eriophyoids, like some other arthropods (e.g., prostigmatic and oribatid mites, springtails, diplurans, pseudoscorpions, pauropods or polyxenids) reproduce by sex dissociation (non-pairing) (Thomas and Zeh 1984; Proctor 1998; Michalska et al. 2010). Males deposit spermatophores on a substrate, while females search for them and, regardless of the presence of males, pick up sperm from spermatophores. Although in many species of non-pairing arthropods the rates of spermatophore production are high (most probably due to uncertainty of sperm uptake), non-pairing males can be prudent in spermatophore investment, adjusting the rate of spermatophore deposition to the presence of potential ‘mates’, as well as competitors and their spermatophores (Witte 1991; Proctor 1992, 1998). A similar phenomenon has also been observed in eriophyoids, in which males increased or decreased spermatophore deposition rate according to the presence of females, males and their spermatophores (Michalska 2000, 2005; Michalska and Studnicki 2013), as well as plant condition (e.g., leaf age, mechanical leaf damage or leaf damage caused by conspecific feeding) (Michalska and Shi 2004).

The object of the study was the vagrant eriophyoid, *Aculops allotrichus* Nalepa. The unique feature of this species is that males engage in many-hour-long solitary or joint-guarding of quiescent female nymphs and deposit spermatophores beside them. Nonetheless, similarly to other eriophyoids, males of *Ac. allotrichus* also deposit spermatophores in the absence of female quiescent nymphs and other conspecifics (Michalska 1999, 2012; Michalska et al. 2010). Males of this species were tested in two situations of predation risk: (1) on leaf arenas with cues left by the phytoseiid mite *Amblyseilus swirskii* (Athias-Henriot), and (2) on leaf arenas with injured conspecifics. Additionally, *Ac. allotrichus* males were tested on leaf arenas with the cues left by yeast-fed acarid mite *Tyrophagus putrescentiae* (Schrank). The purpose of this treatment was to determine whether the simple presence of the cues of the non-enemy mite, about as large as the phytoseiid mite, could affect the spermatophore deposition of *Ac. allotrichus*.

## Materials and methods

The eriophyoid mite *Ac. allotrichus* inhabits leaves of the black locust tree, *Robinia pseudoacacia* L. Leaves infested by eriophyoids were sampled from a black locust tree growing on the campus of the Warsaw University of Life Sciences (Warsaw, Poland). Clean leaves were collected from another black locust tree, which was non-infested by eriophyoids.

Males of *Ac. allotrichus* live up to 22 days under laboratory conditions, and they gradually decrease their spermatophore output with age (K. Michalska, unpubl.). To diminish the variance in spermatophore production among eriophyoids I used *Ac. allotrichus* males that were almost equal in age, ca. 3.5–4 days old for the tests. They were reared from the stage of quiescent nymphs, in isolation from other eriophyoids, for 4 days. Just after the infested leaves of *R. pseudoacacia* were brought to the laboratory, male quiescent nymphs were selected from eriophyoid colonies and put singly onto leaf arenas of rearing cages. Males emerged within 10 h of quiescence. After 2 days, the males were transferred to new cages, in which they were kept for the next 2 days until the experiments started.

To rear the males I used four-chambered cages, each with a leaf arena of 0.65 cm in diameter (for details see Michalska 2005). Experiments were carried out within cages with a single leaf arena of 0.55 cm in diameter. Both one- and four-chambered cages were constructed using fresh and ‘clean’ leaves, non-infested by eriophyoids. As in previous studies (Michalska 2005), the edges of the chamber opening were coated with a thin layer of bees wax and then the hole was closed with a square of dialysis membrane. To seal the closure I additionally pressed the membrane to the edges of the chamber opening with a ring of plasticine. The ring was ca. 3 mm thick and its diameter was similar to the diameter of the chamber hole. The cages were maintained on wet cotton in plastic boxes. Both rearing and experiments were carried out in a plant growth chamber at 80-90 % RH, 26 °C and 16:8 LD photoperiod.

*Amblyseius swirskii* is a generalist predator that originates from the East Mediterranean coast. It is commonly used in a biological control against several pest arthropods including spider mites, eriophyoids, thrips and whiteflies (Park et al. 2010; Lee and Gillespie 2011; Calvo et al. 2015). For this study, *Am. swirskii* was obtained from the commercial product Swirskii-Mite—LD by Koppert Biological Systems. Predatory mites were supplied in paper sachets, mixed with the bran and storage mites.

For the tests, *Am. swirskii* females were fed on *Ac. allotrichus*. The feeding treatment was conducted in detached leaf cages with a leaf arena of 1 cm in diameter (for details see Michalska 2000) in a plant growth chamber at 26 °C, 80–90 % RH, and 16:8 LD photoperiod. As the phytoseiids were commercially fed on storage mites, I first deprived predatory females from food for 48 h on ‘clean’ leaf arenas. Then I transferred the hungry females to arenas made of leaves infested by eriophyoids, where they were maintained in groups of several individuals for three consecutive days until the experiment started. Every morning I provided predators with a new supply of eriophyoids by putting fresh pieces of infested leaves into cage chambers. To be certain that experimental phytoseiids were well satiated with eriophyoids and would leave cues, e.g., faeces, in the experimental cages, the last feeding treatment was performed a few hours before tests began.

The effect of the cues of predatory mites on the spermatophore production of eriophyoids was examined by comparing the spermatophore expenditures of *Ac. allotrichus* on leaf arenas exposed to *Am. swirskii* and on clean leaf arenas (control). I placed two *Am. swirskii* females, previously fed on eriophyoids, into each treatment cage. Treatment and control cages were then maintained in the plant growth chamber for 24 h. After this period, predatory females were removed and the single males of *Ac. allotrichus* were released into all the cages. Males deposited spermatophores for 5 h. After that time the males were removed and the spermatophores were counted.

To examine the impact of the cues of yeast-fed mites on the spermatophore output of eriophyoids, males of *Ac. allotrichus* were tested on leaf arenas both exposed and unexposed to the acarid, *T. putrescentiae*. It is a cosmopolitan mite, common in stored food, house dust, bee and bird nests (Kheradmand et al. 2007). It feeds on a varied diet, including fungi, stored products, plants and insects. *Tyrophagus putrescentiae* used in the tests came from the mite colony that was reared on yeast in the Department of Applied Entomology, Warsaw University of Life Sciences. Similarly as in the experiment with predators, I prepared both the control cages with ‘clean ‘leaf arenas and the treatment cages into each I placed two females of *T. putrescentiae*. After 24 h acarid mites were removed and into all cages single males of *Ac. allotrichus* were released. Males were tested for the next 5 h. Then, they were discarded from the cages and their spermatophores were counted.

To examine whether the presence of damaged conspecifics might affect the spermatophore deposition rate of eriophyoids, I compared the spermatophore output of the single *Ac. allotrichus* males on leaf arenas with ten artificially injured nymphs and on arenas without nymphs (clean leaf arenas). Just before the tests began, the nymphs were chosen within the eriophyoid colony and pierced by a fine needle, which simulated an attack by phytoseiids. To avoid damage to the experimental area, the nymphs were injured on leaves still within the mite colony and then transferred to the experimental cages. The injured individuals were placed evenly along the edge of the arenas, along which males wandered within the cage. Immediately after the placement of the injured nymphs, I released single males of *Ac. allotrichus* into all cages and let them deposit spermatophores for the next 5 h. After that period I removed the males and counted their spermatophores.

In all experiments, males deposited spermatophores under light conditions. Eriophyoids were handled by means of an eye-lash glued to a wooden stick, and the predatory and fungivorous mites, using a fine brush. Mites were handled and observed at 25–100× magnification under an Olympus SZX 12 zoom stereo microscope equipped with an Olympus Highlight 3100 cold light source.

### Statistical analysis

Statistical analysis was performed using statistical software Statgraphics Plus 4.1. Since the data were non-normally distributed, Mann–Whitney *U* tests were applied.

## Results

**Fig. 1:**
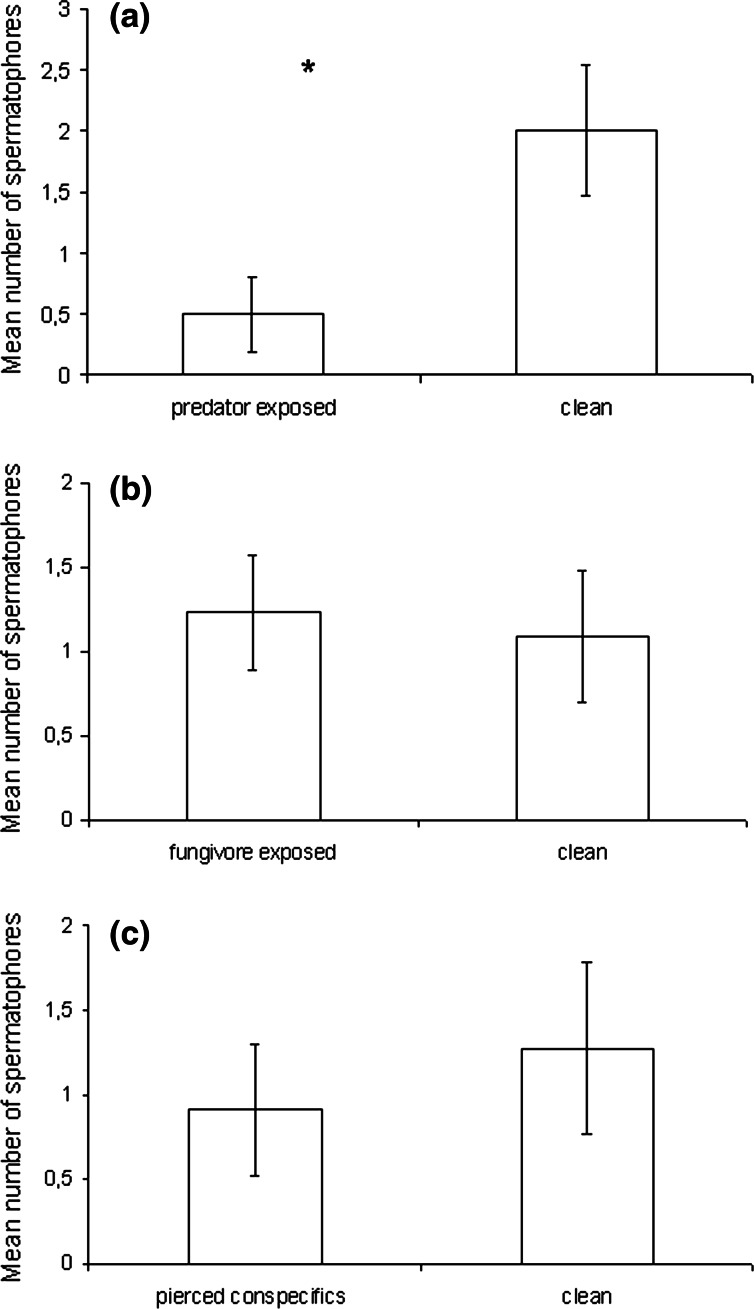
Mean (±SE) number of spermatophores deposited by single males of *Aculops allotrichus* on leaf arenas which were either clean (control) or **a** exposed to predators (*Amblyseius swirskii*), **b** exposed to fungivores (*Tyrophagus putrescentiae*), or **c** provided with pierced nymph conspecifics (Mann–Whitney *U* tests: *P < 0.05)

On the leaf arenas previously exposed to predators, males of *Ac. allotrichus* deposited significantly fewer spermatophores than they did on clean leaves (U = 21, *P* = 0.028; both N = 10) (Fig. 1a). By contrast, they deposited a similar number of spermatophores both on leaf arenas exposed (N = 13) and unexposed (N = 11) to the fungivorous mite *T. putrescentiae* (U = 66.5, *P* = 0.77) (Fig. 1b). Similarly, there were no significant differences in spermatophore output between the males exposed to the presence of pierced conspecifics and the males on clean leaves (U = 52, *P* = 0.58; both N = 11) (Fig. 1c).

## Discussion

In this study, males of *Ac. allotrichus* significantly decreased their spermatophore output on leaf arenas previously exposed to the phytoseiid mites. This inhibitory effect was specific to the presence of the cues of predatory mites, but not to the cues of the fungivorous mite. It is a first study indicating that eriophyoids can respond to predation risk and adjust spermatophore deposition rate according to the presence or absence of the cues of predators. Moreover, this study also shows that predation risk can affect spermatophore output in species reproducing with sex dissociation.

In pairing species, males and/or females can become more conspicuous to predators due to courting, copulation, pre- and post-copulatory struggling, mate guarding, etc. (Travers and Sih 1991; Rowe 1994; Fuller and Berglund 1996; Koga et al. 1998). However, the opposite phenomenon, in which single individuals are more endangered than their coupled counterparts, has been also reported (Gwynne 1989). When searching for mates, animals often become more active and/or have to abandon their hiding places and can therefore be more easily detected (Sakaluk and Belwood 1984; Gwynne 1989; Rowe 1994; Downes 2002; Fowler-Finn et al. 2014). This may also apply to *Ac. allotrichus* males, which, in the absence of female quiescent nymphs, have to distribute their spermatophores in various places on leaves for ‘random’ mates (Michalska and Studnicki 2013). During this time they may increase simultaneously, both their movement and their encounter rate with predators. Just like the newly emergent females of *Ac. allotrichus*, which, if not surrounded by spermatophores, search for them all over the leaf (Michalska 2014). Eriophyoids are much smaller and slower than their potential predators, and after detection, they have almost no chances of escaping predation (Sabelis and Bruin 1996). Therefore, similarly to other phytophagous mites, they should avoid leaf patches with predator cues and also decrease their reproductive activities, e.g., spermatophore deposition, searching for spermatophores, picking up sperm or egg laying on ‘endangered’ leaves. For *Ac. allotrichus* males, the daily spermatophore output is relatively low (Michalska 2011). Thus, it appears to be not only risky for them to stay on leaves occupied by predators but also unprofitable to invest in spermatophores on patches, which may be never visited by potential mates.

As previous studies showed, eriophyoid males can diminish spermatophore deposition rate in various ‘unprofitable’ situations, e.g., in the presence of conspecific males, on leaves without the cues of conspecifics (and potential ‘mates’) or on mechanically injured leaves (Michalska 2000; Michalska and Shi 2004). Behavioral observations of *Cecidophyopsis hendersoni* (Keifer) revealed that on leaves without cues of conspecifics (on which males decreased spermatophore deposition rate), males increased their walking activity and decreased feeding (Michalska and Shi 2004). Thus, in eriophyoid males, the decrease in spermatophore deposition may be a consequence of changes in other activities. Analogically, in spider mites, as suggested by several authors, a decrease in egg production under risk of predation may result from an observed increase in female movement (Oku et al. 2004; Škaloudová et al. 2007; Fernández-Ferrari and Schausberger 2013; Hackl and Schausberger 2014). Moreover, as exhibited by the phytoseiid mite *Neoseiulus cucumeris* (Oudemans), on a patch endangered by another predator, phytoseiid females can retain eggs and delay laying them until they find a safe place (Montserrat et al. 2007; Abad-Moyano et al. 2010). Thus, one cannot exclude that arriving at an unprofitable or endangered patch, eriophyoids also firstly delay deposition of already produced spermatophores, and then increase walking in order to find a new patch at the cost of feeding and further spermatophore production.

Similarly to *Ac. allotrichus* in this study, *Tetranychus urticae* Koch females did not respond to the cues left by *T. putrescentiae* (Grostal and Dicke 1999, 2000). They did not change their behaviour, either, in relation to the other fungivorous mite, *Rhizoglyphus robini* (Claparède) and the pollen-feeding mite, *Orthotydeus caudatus* (Dugès), whereas they did react to the cues of various carnivorous mites, including phytoseiids and parasitic mites (which were not capable of feeding on teranychids). Interestingly, spider mites also responded to the cues of pollen-fed phytoseiids, but it depended on predator species (Grostal and Dicke 2000). As suggested by Grostal and Dicke (2000), a high concentration of protein metabolites in carnivore excreta could be a general cue of predation risk for tetranychids. However, there may be other sources of enemy recognition, e.g., tracking pheromones of predators, from which herbivore mites could obtain more specific information about a predator, e.g., its belonging to the predatory gild or species. In this study, the pellets of eriophyoid-fed *Am. swirskii* (and also yeast-fed *T. putrescentiae*) were frequently present on the experimental leaf arenas. This could have triggered the avoidance behaviour of eriophyoid males and consequently, changes in their spermatophore deposition rate. However, *Am. swirskii* is a ‘generalist’ predator that does not occur in Poland in the wild (see, e.g., Calvo et al. 2015). It has been used in this study because it is commercially available and easy to rear under laboratory conditions. Nonetheless, the response of eriophyoids to this ‘unfamiliar’ phytoseiid might have differed much from that exhibited to native generalist or specialist predators.

Spider mites can also react to indirect information about predation risk, i.e., the presence of injured conspecifics (Grostal and Dicke 1999; Oku et al. 2003). However, as this study revealed, eriophyoid males did not respond to such information and did not change their spermatophore deposition rate in the presence of the pierced conspecifics. The lack of response in *Ac. allotrichus* males might have been connected with the low ‘freshness’ of the cues. In this study, the nymphs were first pierced on a leaf and then transferred to a cage, possibly losing their ‘freshness’ until they were all placed around the leaf arena and a male was released. However, within the eriophyoid colony, I did not observe any disturbance or tendency to escape displayed by the *Ac. allotrichus* males that were in close proximity to the individuals newly damaged by predators (K. Michalska, unpubl.). One explanation of the lack of *Ac. allotrichus* response to the injured conspecifics could be that the latter are simply not a reliable cue of predation risk for the eriophyoids. In colonies, I observed *Ac. allotrichus* individuals accidentally damaged by foraging phytoseiids several times. The predators trampled them, which led to the eriophyoid cuticle breaking off, and the body content flowing out (K. Michalska, unpubl.). As eriophyoids are usually much smaller than all other mites or insects visiting leaves, they may also be accidentally crushed by wandering fungivorous or herbivorous creatures. However, this phenomenon may occur rarely and the lethal risk connected with it may be low. Thus, the presence of injured conspecifics may still not indicate the risk of predation and the necessity to respond.

Recent findings by Fernández-Ferrari and Schausberger (2013) have revealed that female spider mites can vary in their innate sensitivity to threat. Moreover, the sensitivity of spider mites depends on their previous experience with predation risk (Hackl and Schausberger 2014). Thus, to estimate all potential cues of enemy recognition in eriophyoids and their threat sensitivity, many more tests with both eriophyoid males and females and various predator species are needed.
